# Immersive virtual reality as a novel approach to improve social cognition in multiple sclerosis: an EEG-based pilot study

**DOI:** 10.3389/fnins.2026.1812405

**Published:** 2026-04-14

**Authors:** Maria Grazia Maggio, Serena Dattola, Salvatore Bertino, Simona De Salvo, Elvira Gjonaj, Antonino Lombardo Facciale, Luca Pergolizzi, Lilla Bonanno, Federica Impellizzeri, Luana Billeri, Paolo De Pasquale, Angelo Quartarone, Rocco Salvatore Calabrò

**Affiliations:** 1 Neurorehabilitation Unit, IRCCS Centro Neurolesi Bonino-Pulejo, Messina, Italy; 2 Azienda Ospedaliera Papardo, Contrada Papardo, Messina, Italy

**Keywords:** EEG spectral parameterization, immersive virtual reality, multiple sclerosis, neurophysiological biomarkers, neurorehabilitation, social cognition

## Abstract

**Introduction:**

Multiple sclerosis (MS) affects different cognitive domains, including social cognition. Immersive Virtual Reality (VR) may provide a novel rehabilitative approach to treat motor and cognitive symptoms of MS. This exploratory pilot study evaluated the effects of immersive VR rehabilitation on social cognition in MS patients and explored related cortical neurophysiological signatures.

**Methods:**

Seven MS patients underwent immersive VR rehabilitation with the CAREN system (3 sessions/week, approximately 45 min of active training per session, about 1 h including preparation, 8 weeks), while seven healthy controls (HC) did not undergo any intervention. Patients were evaluated at baseline (T0) and post-treatment (T1) with standardized measures of cognitive, emotional, and motor functioning. EEG data were acquired from all participants, and, after artifact removal, spectral parameterization decomposed signals into aperiodic (exponent, offset) and periodic oscillatory components (alpha and beta power). Power spectral density was analyzed using group comparisons and Pearson correlations with neuropsychological measures.

**Results:**

Compared with HC, MS patients showed reduced alpha-band power, mainly over frontal and parieto-occipital regions, whereas aperiodic parameters did not differ between groups. In patients, alpha and beta power correlated with the Positive Emotions Self-Efficacy Scale (alpha: *r* = 0.92, *p* = 0.003; beta: *r* = 0.83, *p* = 0.020). Alpha power is also correlated with RAO SRT–LTS (*r* = 0.85, *p* = 0.016), and beta with EQ-CE (*r* = 0.82, *p* = 0.023). Overall, alpha and beta power were correlated with emotional self-efficacy, balance, memory, and empathy, suggesting that oscillatory markers are potential indicators of clinical outcomes.

**Discussion:**

Rehabilitation via immersive VR has shown promising clinically significant effects in the cognitive, emotional, and motor domains, supported by convergent EEG spectral signatures. Future studies employing predictive modeling approaches will be required to assess their prognostic value.

## 1 Introduction

Social cognition (SC) refers to a set of neurocognitive processes that enable individuals to perceive, interpret, and respond to the intentions, emotions, and behaviors of others (Adolphs, 2009; Goettfried et al., 2025). It encompasses diverse interconnected domains, including emotion recognition, empathy, theory of mind (ToM), and social decision-making, all of which are essential for adaptive interpersonal functioning (Melloni et al., 2014; Mitchell and Phillips, 2015). Among these domains, empathy plays a pivotal role and is commonly divided into cognitive empathy, which involves understanding the mental states of others, and affective empathy, the ability to share in the emotional experiences of others (Shamay-Tsoory, 2011; Decety et al., 2012). These processes rely on a complex and distributed brain network, including the medial prefrontal cortex, temporoparietal junction, and other limbic-related regions such as the anterior cingulate cortex and insula (Apps et al., 2016; Arioli et al., 2018; Krendl and Betzel, 2022). Alterations in these systems have been documented in several neurological and neurodegenerative conditions, in which impairment to the fronto-limbic and temporo-parietal pathways impairs the ability to process social and emotional signals (Melloni et al., 2014; Vandekerckhove et al., 2020). Among these, multiple sclerosis (MS) is increasingly recognized as a disorder that extends beyond motor and sensory impairment, affecting higher-order cognitive and socioemotional functions (Henry et al., 2016; Benedict et al., 2020). MS represents a particularly relevant clinical model for investigating SC impairments, as disease-related structural and functional alterations frequently involve fronto-limbic and temporoparietal networks supporting emotional processing, empathy, and theory of mind. These alterations may lead to deficits in socio-emotional functioning that negatively affect interpersonal relationships and quality of life, highlighting the importance of developing targeted rehabilitation approaches for this domain. Some authors have highlighted that the demyelinating and neurodegenerative processes of MS could influence the networks involved in emotional processing and social understanding, particularly within the prefrontal-limbic circuits (Krause et al., 2009; Kalin, 2019). Consequently, individuals with MS frequently exhibit deficits in emotion recognition, reduced empathy, and impaired ToM skills (Pöttgen et al., 2013; Raimo et al., 2017), which significantly impact interpersonal relationships, psychosocial adjustment, and overall quality of life (Phillips et al., 2011).

Traditional rehabilitation approaches have focused primarily on general motor and cognitive outcomes, often neglecting the socio-cognitive and emotional domains, which are crucial determinants of functional recovery and social participation. In this context, immersive Virtual Reality (VR) has emerged as a promising rehabilitation tool capable of combining scenarios mimicking reality with precise experimental control (Tieri et al., 2018; Maggio et al., 2019; Riva et al., 2019; Maggio et al., 2024a). Immersive VR systems, such as the Computer Assisted Rehabilitation Environment (CAREN), can simulate realistic social scenarios, enhance motivation, embodied experience, and multisensory integration, while promote neural plasticity (Cellini et al., 2022; Impellizzeri et al., 2024; Maggio et al., 2024c,2024b; Wankhede et al., 2025). However, despite these advantages, VR-based rehabilitation may also present some limitations, particularly in neurological populations such as individuals with MS. Factors such as fatigue, sensory overload, or cybersickness may influence patients’ tolerance to immersive environments, highlighting the need for individualized training protocols and careful monitoring of exposure time to ensure safety and treatment effectiveness (Feng et al., 2025). This technology offers a unique opportunity to stimulate SC, engaging the same neural networks implicated in empathy and emotion regulation (Maresca et al., 2020; Krendl and Betzel, 2022; Sievers and Thornton, 2024). To better understand how these immersive experiences impact brain function, the integration of neurophysiological measures such as electroencephalography (EEG) is crucial, as highlighted by previous studies (Mishra et al., 2021; Kim, 2025). EEG offers a non-invasive approach to understanding neurophysiological mechanisms underlying such interventions. Oscillatory brain activity, particularly within the alpha and beta frequency bands, has been associated with attentional control, emotional processing, and empathic engagement (Riels et al., 2022; Codispoti et al., 2023). Understanding the relationship between EEG oscillatory dynamics and behavioral outcomes can therefore contribute to the identification of recovery biomarkers and the optimization of individualized neurorehabilitation programs (Babiloni et al., 2020; Esteves et al., 2025). Although EEG has been increasingly used to characterize neurophysiological alterations in MS, findings related to EEG power spectral density (PSD) remain underexplored and heterogeneous (Facchetti et al., 1994; Leocani et al., 2000b; Cogliati Dezza et al., 2015; Babiloni et al., 2016a). Moreover, only one study has investigated resting-state EEG modifications following a motor-based rehabilitation program in MS (Tramonti et al., 2019). In that study, no correlations were found between changes in spectral power (delta, theta, alpha, beta) and improvements in motor performance, as measured by the Timed Up and Go test.

Overall, these findings suggest that immersive VR rehabilitation, combined with EEG monitoring, could represent an innovative and effective approach to improving socio-cognitive functioning in individuals with MS.

This exploratory pilot study aimed to investigate the potential effects of immersive VR training on social cognition in patients with MS and to explore its neurophysiological correlates through EEG spectral analysis. Specifically, the study pursued two main objectives:

(1) To assess longitudinal changes in cognitive, emotional, and neurophysiological measures (EEG spectral parameters) in MS patients, comparing evaluations conducted before (T0) and after (T1) an immersive VR rehabilitation program;
(2) To compare the spectral features of resting-state EEG in MS patients at baseline with those of healthy controls, to characterize disease-related neurophysiological differences.

Thus, this study aims to explore the potential of immersive VR as a tool for enhancing social cognition and to identify oscillatory biomarkers associated with emotional and cognitive improvement in MS.

## 2 Materials and methods

### 2.1 Study population

This pilot study was conducted between November 2023 and May 2024 at the IRCCS Centro Neurolesi “Bonino-Pulejo” in Messina, Italy. The study involved a total of 14 subjects, including 7 patients with MS and 7 HC, matched for age, sex, and education level. Neurological disability in the MS group was assessed using the Expanded Disability Status Scale (EDSS), a standardized clinical measure widely used to quantify disability in multiple sclerosis. In the present cohort, participants with MS showed a moderate level of disability (mean EDSS = 4.36 ± 0.90). Information on disease-modifying therapies was collected for all MS patients. At the time of the study, patients were receiving stable disease-modifying treatments (ocrelizumab, fingolimod, or natalizumab), as reported in Table 1.

**TABLE 1:** Demographic and clinical characteristics of MS patients and HC.

Variable	MS (*n* = 7)	HC (*n* = 7)	*p*-value
Age (mean ± SD)	41.57 ± 10.20 years	42.29 ± 11.18 years	0.903
Sex (F/M)	3 / 4	3 / 4	1
Disease duration (mean ± SD)	15.43 ± 10.21 years	–	NA
Pharmacological treatment	Ocrelizumab (*n* = 3)	–	NA
	Fingolimod (*n* = 2)		
	Natalizumab (*n* = 2)		
EDSS (T0) (mean ± SD)	4.36 ± 0.90	–	–
EDSS (T1) (mean ± SD)	4.36 ± 0.90	–	–
BBS (T0) (mean ± SD)	47.29 ± 7.23	–	–
BBS (T1) (mean ± SD)	50.00 ± 7.09	–	–

EDSS-T0, Expanded Disability Status Scale, baseline; EDSS-T1, Expanded Disability Status Scale, post-intervention; BBS-T0, Berg Balance Scale, baseline; BBS-T1, Berg Balance Scale, post-intervention.

Inclusion criteria for the MS group were: (i) a confirmed diagnosis of relapsing-remitting MS according to the revised 2017 McDonald criteria (Thompson et al., 2018); (ii) stable clinical condition without relapses or corticosteroid therapy in the previous three months, and (iii) the ability to participate in the virtual reality training sessions.

Exclusion criteria included severe cognitive impairment (Mini-Mental State Examination < 10), as individuals with severe cognitive deficits may have difficulty reliably cooperating with neuropsychological assessments and EEG recordings (Tombaugh and McIntyre, 1992), severe psychiatric or neurological comorbidities, visual or hearing impairments that could interfere with VR participation, and contraindications to EEG recording.

Healthy control group participants were recruited from the local community and screened for neurological, psychiatric, or systemic conditions that could impair cognitive performance.

All participants were right-handed and had normal or corrected-to-normal vision.

### 2.2 Ethics

The study was conducted in accordance with the principles of the Declaration of Helsinki. All participants provided written informed consent prior to enrollment. The research forms part of a larger multi-year project coordinated by the IRCCS Centro Neurolesi “Bonino-Pulejo” and registered on ClinicalTrials.gov (ID: NCT07066137). Ethical approval was obtained from the local Ethics Committee (Protocol code: IRCCSME 47/2023).

### 2.3 Outcome measure

MS patients completed a comprehensive battery of clinical, cognitive, and socioemotional assessments at T0 and T1 (Table 2). The outcome measures are covered four main domains: SC and emotional processing, alexithymia, emotional self-efficacy, and cognitive-motor functioning.

**TABLE 2:** Neuropsychological and clinical assessment tools and reference cut-offs used for MS patients.

Domain	Assessment tool	Main subscales/components	Cut-off/interpretation	References
Social cognition	Empathy Quotient (EQ)	Cognitive Empathy (EQ-CE); Emotional Reactivity (EQ-ER); Social Skills (EQ-SS); Total score	EQ Total < 30 = low empathy (clinical range); 30–50 = below average	Baron-Cohen and Wheelwright, 2004
Emotional processing/awareness	Toronto Alexithymia Scale (TAS-20)	Difficulty Identifying Feelings (TAS-IF); Difficulty Describing Feelings (TAS-DF); Externally Oriented Thinking (TAS-EO); Total score (TAS-TOT)	TAS-20 Total ≥ 61 = alexithymia; 52–60 = possible alexithymia	Bagby et al., 2014
Emotional self-efficacy	Emotional Self-Efficacy Scale (ESES)	Regulation of Positive Emotions; Regulation of Negative Emotions	No fixed clinical cut-off; higher scores = greater perceived self-efficacy	Caprara et al., 2008
Cognitive functioning	Rao’s Brief Repeatable Battery of Neuropsychological Tests (BRB-N)	SRT (LTS, CLTR, D); SPART (Immediate, Delayed); SDMT; PASAT (2, 3 s); WLG	SRT-LTS (cut-off: 23.3); SRT-CLTR (15.5); SRT-D (4.9); SPART (12.7); SPART-D (3.6); SDMT (37.9); PASAT 3 (28.4); PASAT 2 (17.1); WLG (17.0 men/women). Scores below cut-off indicate impaired performance	Tedone et al., 2024
Motor/balance function	Berg Balance Scale (BBS)	Total score assessing postural control and fall risk	BBS ≤ 45 = increased fall risk	Berg et al., 1992

SC was evaluated using the Empathy Quotient (EQ), which measures three core components of empathic functioning: Cognitive Empathy (EQ-CE), which reflects the ability to understand the mental states of others; Emotional Reactivity (EQ-ER), which indicates affective reactivity to the emotions of others; and Social Skills (EQ-SS), which measures the ability to manage social interactions (Baron-Cohen and Wheelwright, 2004).

Emotional awareness and regulation were assessed using the Toronto Alexithymia Scale (TAS-20), a standardized self-report questionnaire that measures three aspects of alexithymia: difficulty identifying emotions (TAS-IF), difficulty describing emotions (TAS-DF), and externally oriented thinking (TAS-EO), along with a total score (TAS-TOT) (Bagby et al., 2014).

Participants’ perceived ability to manage emotions was measured using the Emotional Self-Efficacy Scale, which separately assesses effectiveness in regulating positive and negative emotions (Emotional Self-Efficacy—Positive/Negative). Higher scores indicate greater confidence in one’s emotional regulation abilities (Caprara et al., 2008).

Cognitive performance was assessed using the Rao Brief Repeatable Battery of Neuropsychological Tests (BRB-N), widely used in MS research (Tedone et al., 2024). The battery includes: Selective Reminding Test (SRT-LTS, SRT-CLTR, SRT-D) for learning and verbal memory; Spatial Recall Test (SPART, SPART-D) for visuospatial memory; Symbol Digit Modalities Test (SDMT) for processing speed; Pacing Auditory Serial Addition Test (PASAT 2 and PASAT 3) for attention and working memory; and Word List Generation (WLG) for verbal fluency and executive functions.

Functional balance was assessed using the Berg Balance Scale (BBS), a validated clinical measure of postural control, and fall risk (Berg et al., 1992).

All measures were collected at two time points (T0, T1) under standardized conditions. The comprehensive assessment was designed to capture multidimensional changes in cognitive, emotional, and motor domains following immersive VR training.

Healthy subjects were assessed by EEG only.

### 2.4 EEG data acquisition and processing

EEG activity was recorded from all participants (7 MS patients at T0 and T1 and 7 HC) during resting state with eyes closed. EEG signals were collected using a Brain-Quick System (Micromed) with 19 electrodes positioned according to the international 10–20 system (Fp1, Fp2, F7, F8, F3, F4, Fz, T3, T4, C3, C4, Cz, P3, P4, Pz, T5, T6, O1, O2). Although different electrode configurations or reduced electrode sets can be adopted depending on the study design (Ding et al., 2025), the full 19-channel 10–20 montage represents the conventional configuration used in clinical EEG practice (Seeck et al., 2017). Signals were acquired in a differential montage, referenced to linked earlobes. Electrode impedances were kept below 5 kΩ. Data were acquired at a sampling rate of 1024 Hz. EEG preprocessing was carried out using EEGLAB (v2025.1.0) and custom MATLAB (R2023b) scripts. A notch filter was applied at 50 Hz to reduce line noise and improve the signal-to-noise ratio. Data were then band-pass filtered between 0.5 and 40 Hz and downsampled to 256 Hz. Bad channels and bad segments were automatically removed before running the ICA. The removed channels were then interpolated, and the data were finally average-referenced. All preprocessing steps were performed offline. PSD was computed using Welch’s method, with 2-second Hamming windows and 50% overlap, to obtain a stable estimate of frequency content for each electrode. Spectral parameterization was then performed using the FOOOF algorithm (Donoghue et al., 2020). Traditional EEG spectral analyses typically quantify power within predefined frequency bands, but they do not distinguish between true oscillatory activity and broadband aperiodic components of the neural power spectrum. Consequently, variations in the aperiodic background activity may lead to misinterpretations of oscillatory power changes. The FOOOF approach addresses this limitation by modeling the neural power spectrum as the sum of two distinct components: an aperiodic component, which represents the “1/f” background activity, and periodic components, which correspond to oscillatory peaks that rise above this background. The aperiodic components derive from non-rhythmic EEG activity. Specifically, the exponent reflects the general decay of power with increasing frequency and is thought to estimate overall excitatory-inhibitory balance in cortical circuits, while the offset reflects a uniform shift of power across frequencies (Gao et al., 2017; Donoghue et al., 2020). Importantly, recent clinical research suggests that features of the aperiodic component may represent potential electrophysiological biomarkers across several neurological and psychiatric conditions, highlighting the relevance of separating periodic and aperiodic activity when interpreting EEG spectral changes (Pani et al., 2022).

The functional form is:


$$L\,\left(F\right)=b-\log\left(k+F_{\chi }\right)$$

where *b* is the offset (overall vertical translation), *k* is the “knee” parameter (optional, representing a bend in the spectrum), and χ is the exponent reflecting the slope of the aperiodic component in log–log space. In contrast, the periodic components capture rhythmic oscillations, such as alpha or beta activity, linked to network synchronization (Buzsáki and Draguhn, 2004). Each peak *n* is modeled by a Gaussian function:


$$G\,{\left(F\right)}_{n}=a*\exp\left(\left(-{\left(F-c\right)}^{2}\right)/\left(2*w^{2}\right)\right)$$

where *a* is the peak height (power above the aperiodic background), *c* is the center frequency of the peak, *w* is the width (bandwidth) of the Gaussian, and *F* is the array of frequency values. The full model of the neural power spectra (NPS) therefore becomes:


$$N\,P\,S\,\left(F\right)=L\,\left(F\right)+G\,{\left(F\right)}_{n}$$

For each participant and electrode, the FOOOF fitting returned two aperiodic parameters (exponent and offset) and periodic peaks for alpha (8–13 Hz) and beta (13–30 Hz) bands. The FOOOF model was initialized with a maximum of six peaks, a minimum peak threshold of 0.5, and peak width limits between 0.5 and 12 Hz. These parameters were chosen to allow the identification of multiple oscillatory components, while minimizing the risk of detecting spurious peaks.

Average band power values were computed across electrodes for group-level analyses.

### 2.5 Procedures

After providing written informed consent, participants were submitted to baseline assessments (T0). These included cognitive, motor, and socio-emotional assessments, as well as resting-state EEG recordings. Only the MS group participated in the immersive VR rehabilitation program, while healthy controls underwent only baseline EEG acquisition.

The rehabilitation program consisted of 20 sessions, delivered three times per week for approximately 8 weeks, with each session lasting about 45 min of active training (approximately 1 h including preparation and breaks). Training was delivered using the CAREN immersive system, a multisensory platform based on motion capture that integrates a dynamic platform, motion tracking, and multiple synchronized projection screens.

The immersive environment enabled real-time interaction with virtual activities designed to simultaneously stimulate cognitive, motor, and social areas. Four different virtual scenarios were used, focusing primarily on dual-task and socio-emotional training. Dual-task exercises combined cognitive and motor demands to improve attentional flexibility and executive control, while socio-emotional scenarios included social stimuli, such as interactive environments (e.g., a virtual city with a pizza cart), aimed at improving emotional processing and perspective-taking. Task difficulty and sensory feedback were progressively adapted to each patient’s performance to maintain engagement and ensure optimal challenge.

During each session, task complexity and environmental feedback were progressively adapted based on individual performance to maintain engagement and ensure adaptive challenge. Visual and auditory feedback were provided in real time to facilitate self-monitoring and reinforce correct execution.

At the end of the rehabilitation program, MS patients underwent post-intervention assessments (T1) identical to those administered at baseline, which included cognitive, emotional, and motor assessments, as well as EEG recordings. All sessions were supervised by qualified neuropsychologists and physiotherapists with experience in neurorehabilitation. Therapists responsible for the intervention were not involved in outcome evaluations to minimize bias.

### 2.6 Statistical analysis

Between-group comparisons (MS at T0 vs. HC) were performed using independent-samples two-tailed *t*-tests, whereas within-patient longitudinal comparisons (T0 vs. T1) were conducted using paired-samples two-tailed *t*-tests. All *t*-tests were computed pairwise for homologous electrodes. *P*-values were corrected for multiple comparisons using the false discovery rate (FDR, *p* < 0.05). Within the MS group, Pearson correlation coefficients were computed between EEG band power and the neuropsychological measures collected at baseline and post-intervention. All statistical analyses were conducted using MATLAB (R2023b).

## 3 Results

### 3.1 Demographic and clinical characteristics

Demographic and clinical characteristics of the MS patients and HC are summarized in Table 1. The two groups were comparable in terms of age and sex distribution. In the MS group, disability levels remained stable across the intervention period, as reflected by identical EDSS scores at T0 and T1. No pharmacological treatment was present in the healthy control group.

### 3.2 MS (T0) vs. MS (T1)

#### 3.2.1 Neuropsychological assessment

Neuropsychological performance at T0 and T1 in the MS group is reported in Table 3. Paired-sample *t*-tests (two-tailed) were conducted to assess longitudinal changes. Since the sample size was limited (*n* = 7), *p*-values were not corrected for multiple comparisons, consistent with exploratory pilot-study designs. Effect sizes (Cohen’s *d*) and their corresponding 95% confidence intervals (CI) were also computed to quantify the magnitude of the observed changes. A statistically significant improvement was observed in the SRT-LTS score (*p* = 0.011), reflecting enhanced long-term storage verbal memory following the VR-based rehabilitation program. All other cognitive, emotional, and motor measures showed no significant longitudinal variations.

**TABLE 3:** Neuropsychological test scores for MS patients at baseline (T0) and post-intervention (T1).

Test	T0 (mean ± SD)	T1 (mean ± SD)	*p*-value	Cohen’s *d*	95% CI
EQ-CE	4.57 ± 1.81	5.71 ± 2.63	0.103	0.44	[−0.15, 1.27]
EQ-ER	5.86 ± 2.85	6.00 ± 1.73	0.890	0.05	[−0.96, 1.09]
EQ-SS	6.14 ± 1.21	5.86 ± 2.54	0.715	−0.12	[−1.08, 0.76]
TAS-DF	12.71 ± 4.96	10.57 ± 4.65	0.200	−0.39	[−1.29, 0.30]
TAS-IF	14.29 ± 4.31	13.57 ± 4.39	0.786	−0.14	[−1.60, 1.23]
TAS-EO	17.57 ± 6.83	20.86 ± 6.04	0.325	0.44	[−0.63, 1.77]
TAS-TOT	44.57 ± 6.85	45.00 ± 11.60	0.928	0.04	[−1.11, 1.21]
Emotional self-efficacy (positive emotions)	29.14 ± 6.07	28.29 ± 6.87	0.356	−0.11	[−0.48, 0.19]
Emotional self-efficacy (negative emotions)	24.00 ± 5.39	23.57 ± 5.86	0.802	−0.07	[−0.80, 0.63]
SRT-LTS	34.95 ± 14.09	51.38 ± 9.10	0.011*	1.20	[0.29, 2.75]
SRT-CLTR	29.83 ± 14.57	36.41 ± 19.04	0.293	0.34	[−0.42, 1.28]
SPART	15.08 ± 5.99	18.65 ± 7.78	0.160	0.45	[−0.27, 1.41]
SDMT	38.17 ± 11.26	37.59 ± 13.59	0.712	−0.04	[−0.35, 0.24]
PASAT 3	35.95 ± 15.07	40.24 ± 13.61	0.262	0.26	[−0.28, 0.95]
PASAT 2	29.21 ± 9.53	35.21 ± 14.46	0.071	0.43	[−0.07, 1.15]
SRT-D	7.27 ± 1.77	8.27 ± 1.93	0.218	0.47	[−0.41, 1.61]
SPART-D	6.49 ± 3.13	6.63 ± 2.78	0.884	0.04	[−0.72, 0.83]
WLG	25.73 ± 8.24	27.16 ± 6.90	0.587	0.16	[−0.60, 1.02]

EQ-CE, Empathic Concern; EQ-ER, Emotional Reactivity; EQ-SS, Social Skills; TAS-DF, Difficulty Describing Feelings; TAS-IF, Difficulty Identifying Feelings; TAS-EO, Externally-Oriented Thinking; TAS-TOT, Toronto Alexithymia Scale Total; Emotional Self-Efficacy—Positive Emotions; Emotional Self-Efficacy—Negative Emotions; SRT-LTS, Selective Reminding Test—Long-Term Storage; SRT-CLTR, Selective Reminding Test—Consistent Long-Term Retrieval; SPART, Spatial Recall Test; SDMT, Symbol Digit Modalities Test; PASAT-3, Paced Auditory Serial Addition Test-3 seconds; PASAT-2, Paced Auditory Serial Addition Test-2 s; SRT-D, Selective Reminding Test—Delayed; SPART-D, Spatial Recall Test—Delayed; WLG, Word List Generation.

*Indicates statistically significant differences (*p* < 0.05).

#### 3.2.2 Clinical significance of cognitive, emotional, and social-cognitive changes

Although most p-values did not reach statistical significance due to the limited sample size (*n* = 7), several outcomes demonstrated changes that approached or exceeded established minimal clinically important differences (MCID) (Draak et al., 2019) thereby supporting the potential clinical relevance of the observed effects (Table 4).

**TABLE 4:** Clinically meaningful change (MCID) thresholds and observed changes in MS patients.

Domain	Outcome measure	MCID/Clinically meaningful threshold	Observed change (T1–T0)	Clinical interpretation	Key references
Verbal learning and memory	SRT-LTS	MCID ≈ +10–15% improvement in verbal learning	+**16.43 points (≈ +47%)**	Exceeds MCID; large improvement in long-term verbal storage	Boringa et al., 2001
Working memory and attention	PASAT-2	Clinically meaningful change = +10–15%	**+6 points (≈ +20.5%)**	Clinically meaningful despite *p* > 0.05	
Working memory and attention	PASAT-3	Clinically meaningful = +10–15%	**+4.29 points (≈ +12%)**	Meets MCID range	
Processing speed	SDMT	MCID = +4 points	−0.58 points	No clinically meaningful change	Benedict et al., 2020
Visuospatial memory	SPART	No fixed MCID; ≥ 10–15% considered relevant	+**3.57 points (≈ +24%)**	Clinically coherent improvement	Boringa et al., 2001
Executive function/verbal fluency	WLG	No formal MCID; 10–20% improvement considered relevant	+*1.43 points (≈ +5.6%)*	Small but coherent improvement	
Cognitive empathy	EQ-CE	No MCID; small (5–10%) changes can be meaningful	+**1.14 points (≈ +25%)**	Notable improvement in cognitive empathy	Pöttgen et al., 2013
Emotional reactivity	EQ-ER	As above	*+0.14 points (≈ +2.3%)*		
Social skills	EQ-SS	As above	−0.28 points (≈ 4.6%)	No improvement	Raimo et al., 2017
Difficulty identifying feelings	TAS-IF	Decreases of 3–5 points considered meaningful	−0.72 points (≈ 5%)	Below meaningful threshold	Bagby et al., 2014
Difficulty describing feelings	TAS-DF	Decreases of 3–5 points meaningful	−**2.14 points (≈ 16.8%)**	Trend toward improvement	
Externally oriented thinking	TAS-EO	Same threshold	+3.29 points (≈ 18.7%)	No improvement	
Alexithymia total score	TAS-TOT	Decrease ≥ 5 points considered meaningful	+0.43 points (≈ +1%)	No improvement	
Positive emotional self-efficacy	ESES-positive	No MCID; small absolute changes meaningful	−0.85 points (≈ +2.9%)	Slight reduction	Caprara et al., 2008
Negative emotional self-efficacy	ESES-negative	Same	−0.43 points (≈ 1.8%)	No improvement	

EQ-CE, Empathy Quotient-Empathic Concern; EQ-ER, Empathy Quotient-Emotional Reactivity; EQ-SS, Empathy Quotient-Social Skills; TAS-DF, Toronto Alexitimia Test-Difficulty Describing Feelings; TAS-IF, Toronto Alexitimia Test-Difficulty Identifying Feelings; TAS-EO, Toronto Alexitimia Test-Externally-Oriented Thinking; TAS-TOT, Toronto Alexithymia Scale Total; Emotional Self-Efficacy—Positive Emotions; Emotional Self-Efficacy—Negative Emotions; SRT-LTS, Selective Reminding Test—Long-Term Storage; SRT-CLTR, Selective Reminding Test—Consistent Long-Term Retrieval; SPART, Spatial Recall Test; SDMT, Symbol Digit Modalities Test; PASAT-3, Paced Auditory Serial Addition Test-3 seconds; PASAT-2, Paced Auditory Serial Addition Test-2 s; SRT-D, Selective Reminding Test—Delayed; SPART-D, Spatial Recall Test—Delayed; WLG, Word List Generation. Values in the “Observed Change (T1–T0)” column are presented as captions in italics, with the absolute change highlighted in bold. Percentages indicate the relative variation from baseline.

In the cognitive domain, the increase in SRT-LTS scores exceed the MCID reported for verbal learning in MS cohorts, as established in the foundational validation work of the Brief Repeatable Battery (Boringa et al., 2001). Likewise, the magnitude of change in PASAT-2 performance fell within the 10–15% variation typically regarded as clinically meaningful for assessing working memory and sustained attention within the MS Functional Composite framework (Boringa et al., 2001).

Beyond cognitive performance, socio-emotional outcomes also showed clinically coherent trends. Variations across EQ subscales, cognitive empathy, emotional reactivity, and social skills, were consistent with prior evidence demonstrating that even modest absolute score changes may reflect meaningful shifts in socio-emotional functioning in MS, given the sensitivity of these measures to disease-related alterations in affective and interpersonal processing (Pöttgen et al., 2013; Raimo et al., 2017). Similarly, reductions in TAS-20 dimensions related to difficulty identifying and describing emotions, together with stable-to-improved emotional self-efficacy scores, align with established psychometric literature indicating that small score changes in alexithymia and emotion-regulation scales can correspond to measurable improvements in emotional awareness and adaptive regulation (Caprara et al., 2008; Bagby et al., 2014).

Taken together, these MCID-referenced indicators highlight that, despite the limited statistical power inherent to the present pilot sample, several domains exhibited changes of a magnitude consistent with clinically meaningful improvement. This pattern underscores the potential therapeutic impact of immersive VR interventions and warrants confirmation in adequately powered future trials.

#### 3.2.3 EEG power analysis

In this pilot study, we investigated whether resting-state EEG derived measures of periodic and aperiodic activity changed after VR-based rehabilitation protocols.

**FIGURE 1:**
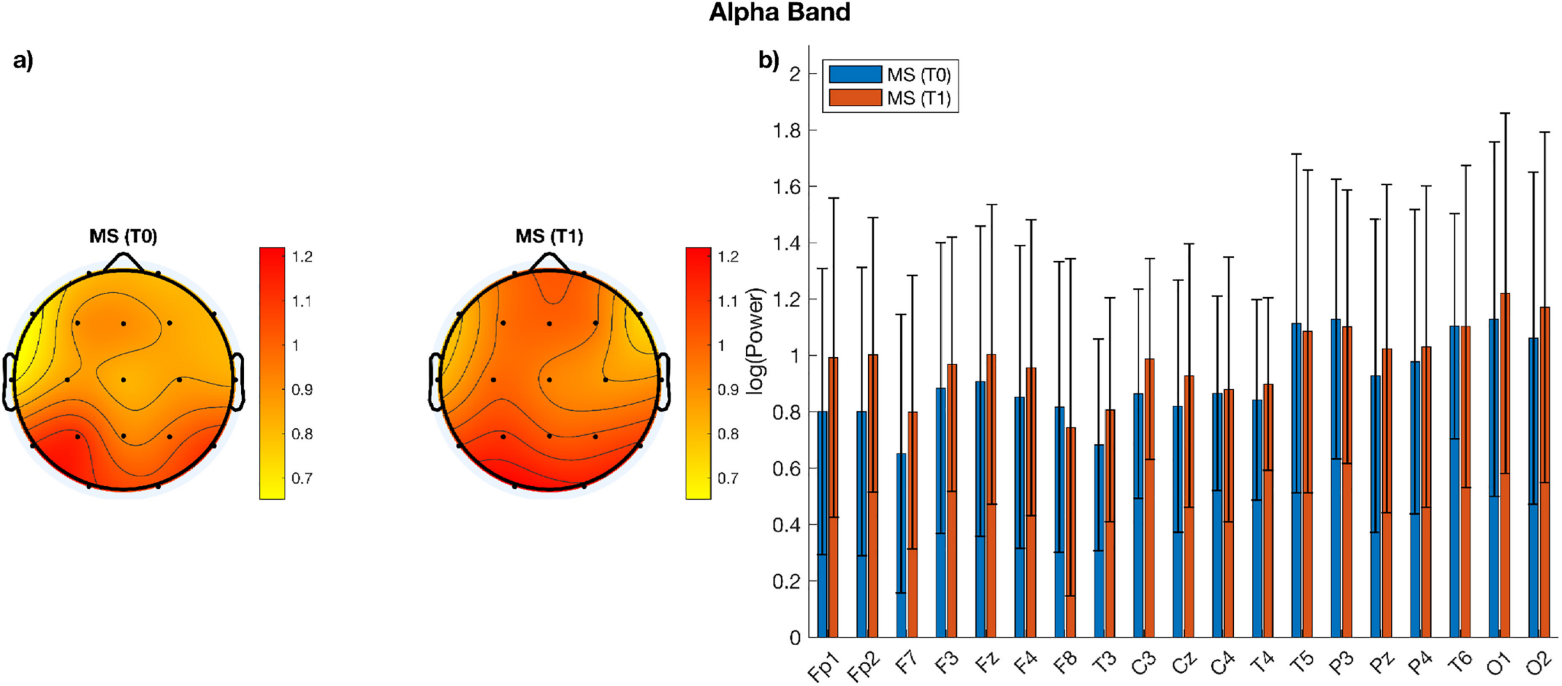
**(a)** Topographic plots of alpha power for MS patients before (T0) and after (T1) treatment; **(b)** bar plots of alpha power per electrode and group. There are no statistically significant differences between groups.

**FIGURE 2:**
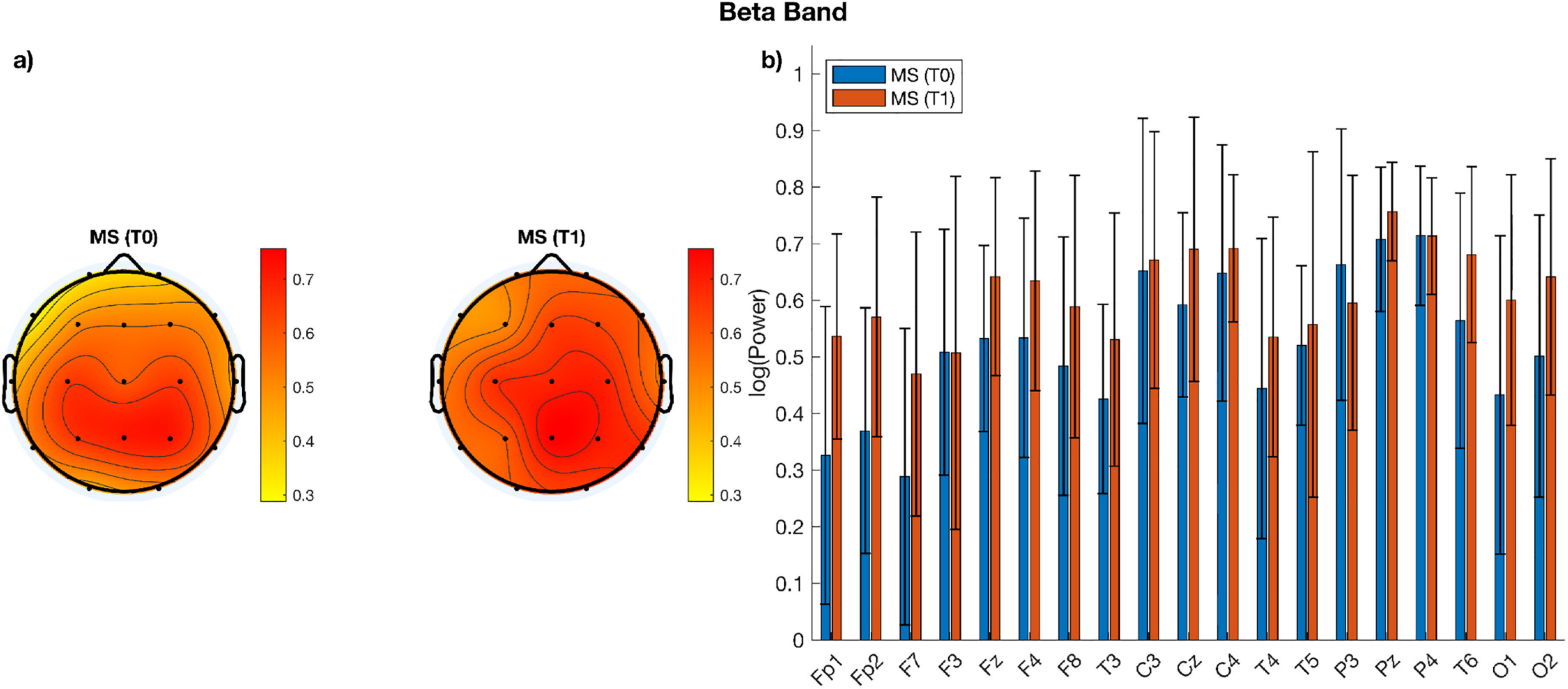
**(a)** Topographic plots of beta power for MS patients before (T0) and after (T1) treatment; **(b)** bar plots of beta power per electrode and group. There are no statistically significant differences between groups.

**FIGURE 3:**
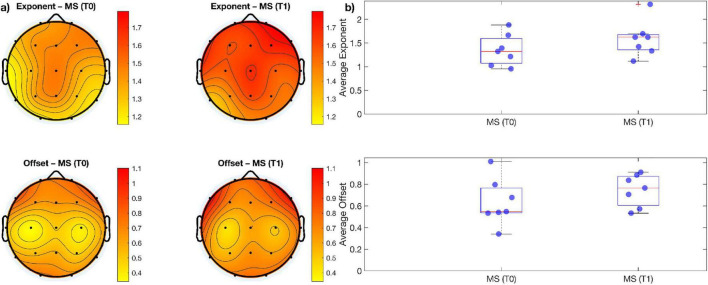
**(a)** Topographic maps showing the spatial distribution of mean exponent and offset values for each group of MS; **(b)** boxplots of mean exponents and offset values across all electrodes. There are no statistically significant differences for both exponents and offsets between groups.

When comparing measures of alpha, beta power over and above the aperiodic component across electrodes using paired sampled *t*-tests, no significant difference emerged between the two time-points (Figures 1, 2). Similarly, no significant change was observed when comparing aperiodic parameters (i.e., exponent and offset) between the pre- and post- treatment period (Figure 3).

### 3.3 MS vs. HC

#### 3.3.1 EEG Power Analysis

**FIGURE 4:**
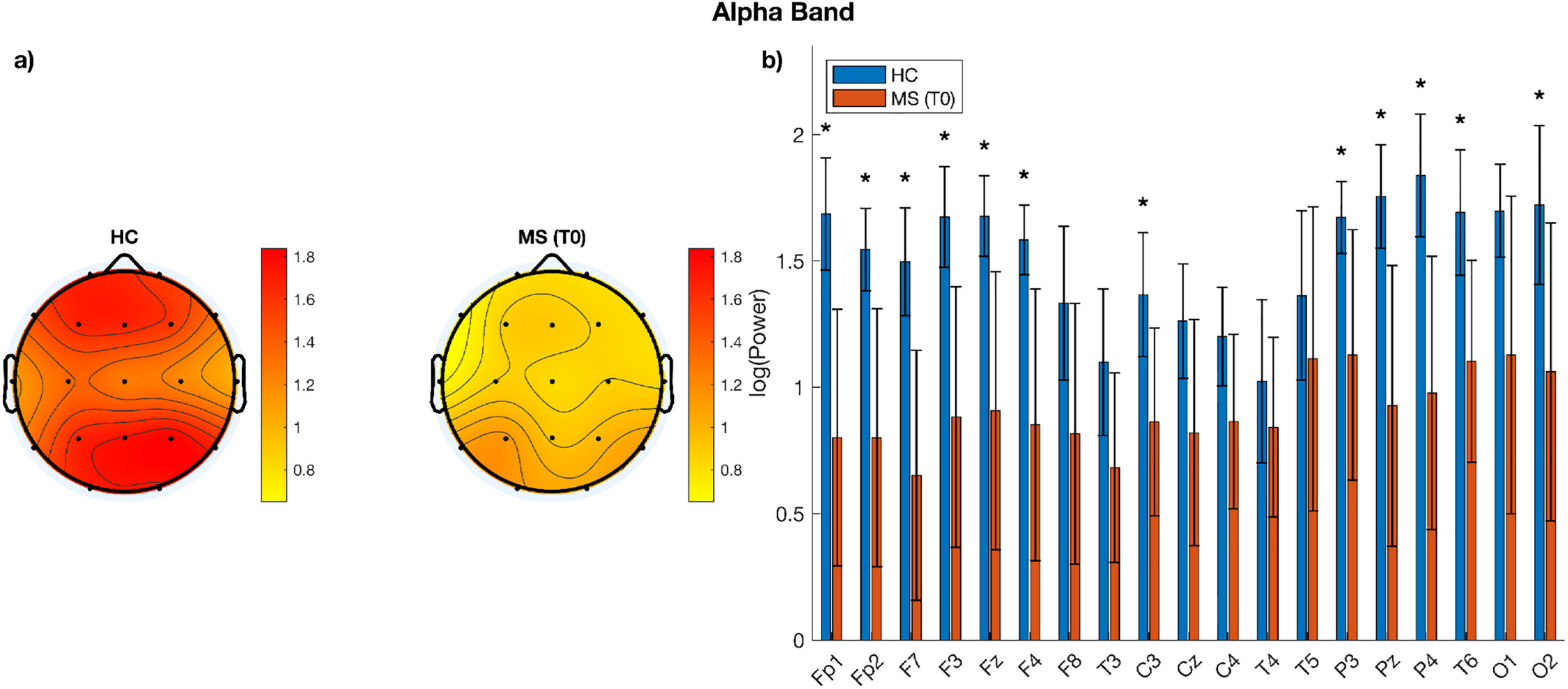
**(a)** Topographic plots of alpha power for each group; **(b)** bar plots of alpha power per electrode and group. Asterisks indicate statistically significant differences between HC and MS (*p* < 0.05).

**FIGURE 5:**
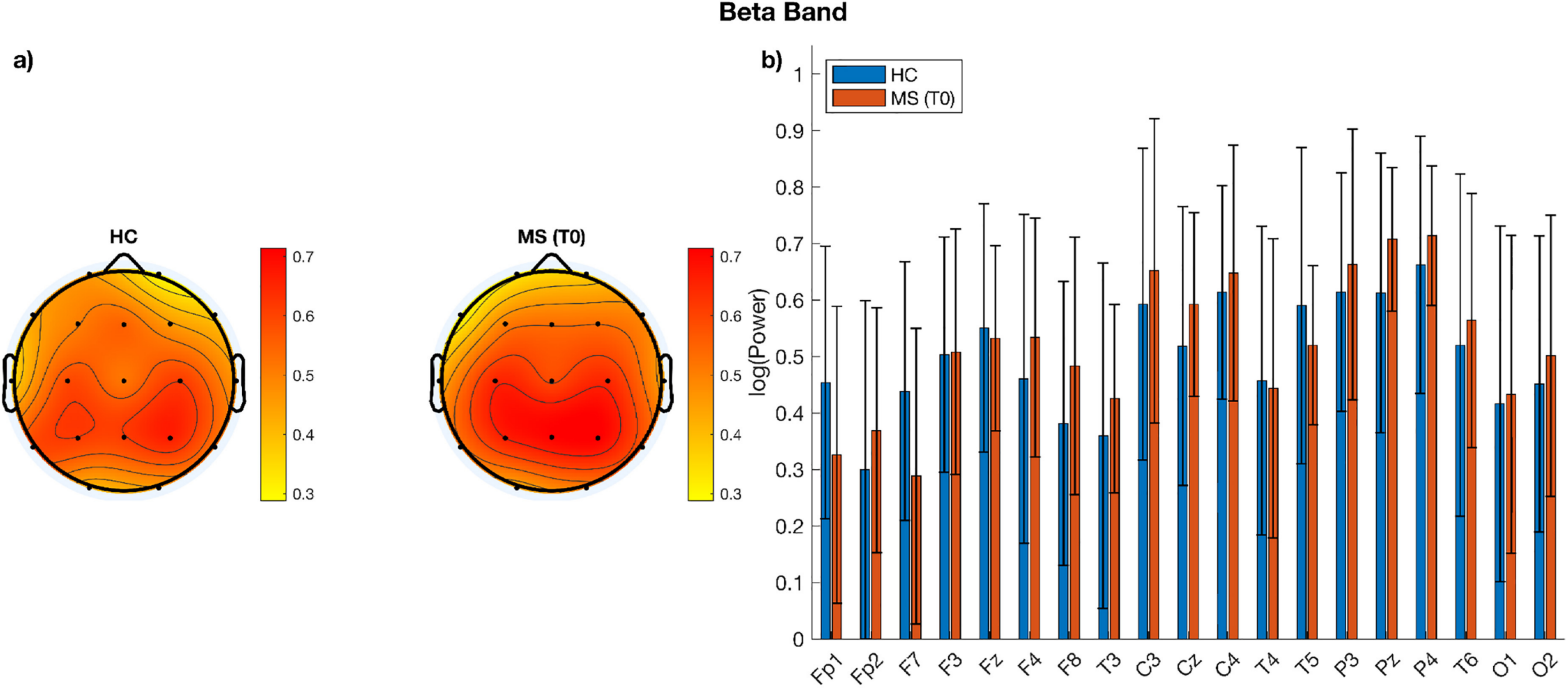
**(a)** Topographic plots of beta power for each group; **(b)** bar plots of beta power per electrode and group. There are no statistically significant differences between HC and MS.

**FIGURE 6:**
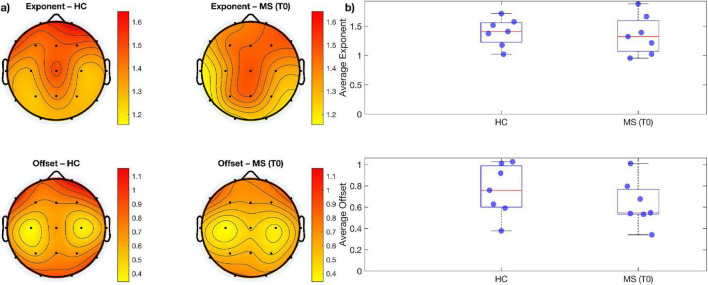
**(a)** Topographic maps showing the spatial distribution of mean exponent and offset values for each group (HC and MS); **(b)** boxplots of mean exponents and offset values across all electrodes. There are no statistically significant differences for both exponents and offsets between groups.

Parameterization of power spectra revealed differences between MS patients and healthy individuals; Specifically, when comparing cortical spectral parameters of patients before undergoing VR rehabilitation to those of healthy controls at baseline, among MS cohort, alpha power showed different topographical distribution when visually compared to healthy individuals with lower power across parieto-occipital and frontal channels (Figure 4a). Such topographical differences were further supported by our channel-wise paired *t*-test showing that alpha power was significantly lower (*p* < 0.05, corrected for multiple comparison) in the MS group over frontal (Fp1, Fp2, F3, F4, F7, Fz), central (C3, Pz) and parieto-occipital electrodes (P3, P4, T6, O2) (Figure 4b). To further quantify the magnitude of these group differences, effect sizes (Cohen’s d) and their 95% CI were computed for each electrode. Large effect sizes were observed across most channels, particularly over frontal and parietal regions, closely matching the spatial distribution of statistically significant electrodes and indicating higher alpha power in HC compared with MS patients. See Table 5 for *p*-values adjusted for multiple comparisons using the FDR method together with the corresponding effect size estimates. On the other hand, beta power was higher over bilateral sensorimotor regions in MS patients in respect to healthy controls (Figure 5a). Despite the differences noticeable in the topographic distribution of beta power, no statistically significant differences were found when performing statistical analysis at single-channel level (Figure 5b). Concerning differences of aperiodic spectral parameters, both exponent and offset showed similar topographic distribution across the scalp between MS patients and healthy individuals (Figure 6a). Channel-level statistics did not show any significant difference between the two groups either for the aperiodic exponent or the offset parameters (Figure 6b).

**TABLE 5:** FDR-corrected p-values from t-tests comparing alpha power between Healthy Controls (HC) and patients with Multiple Sclerosis (MS) at T0, together with effect size estimates (Cohen’s *d*) and their 95% confidence intervals (CI) for each electrode.

Alpha band			
Electrode	*p*-value	Cohen’s d	95% CI
Fp1	0.021*	2.11	[0.80, 3.38]
Fp2	0.021*	1.84	[0.59, 3.03]
F7	0.021*	2.08	[0.77, 3.33]
F3	0.021*	1.90	[0.64, 3.10]
Fz	0.022*	1.78	[0.55, 2.96]
F4	0.023*	1.74	[0.52, 2.92]
F8	0.058	1.14	[0.04, 2.20]
T3	0.058	1.16	[0.06, 2.23]
C3	0.025*	1.50	[0.33, 2.62]
Cz	0.058	1.17	[0.07, 2.23]
C4	0.059	1.12	[0.03, 2.18]
T4	0.356	0.50	[−0.51, 1.49]
T5	0.361	0.48	[−0.53, 1.47]
P3	0.044*	1.40	[0.25, 2.50]
Pz	0.021*	1.85	[0.60, 3.05]
P4	0.021*	1.92	[0.66, 3.14]
T6	0.021*	1.66	[0.46, 2.81]
O1	0.061	1.15	[0.05, 2.21]
O2	0.044*	1.31	[0.18, 2.40]

*p < 0.05.

### 3.4 Correlations analysis between spectral parameters and scores measuring SC

**FIGURE 7:**
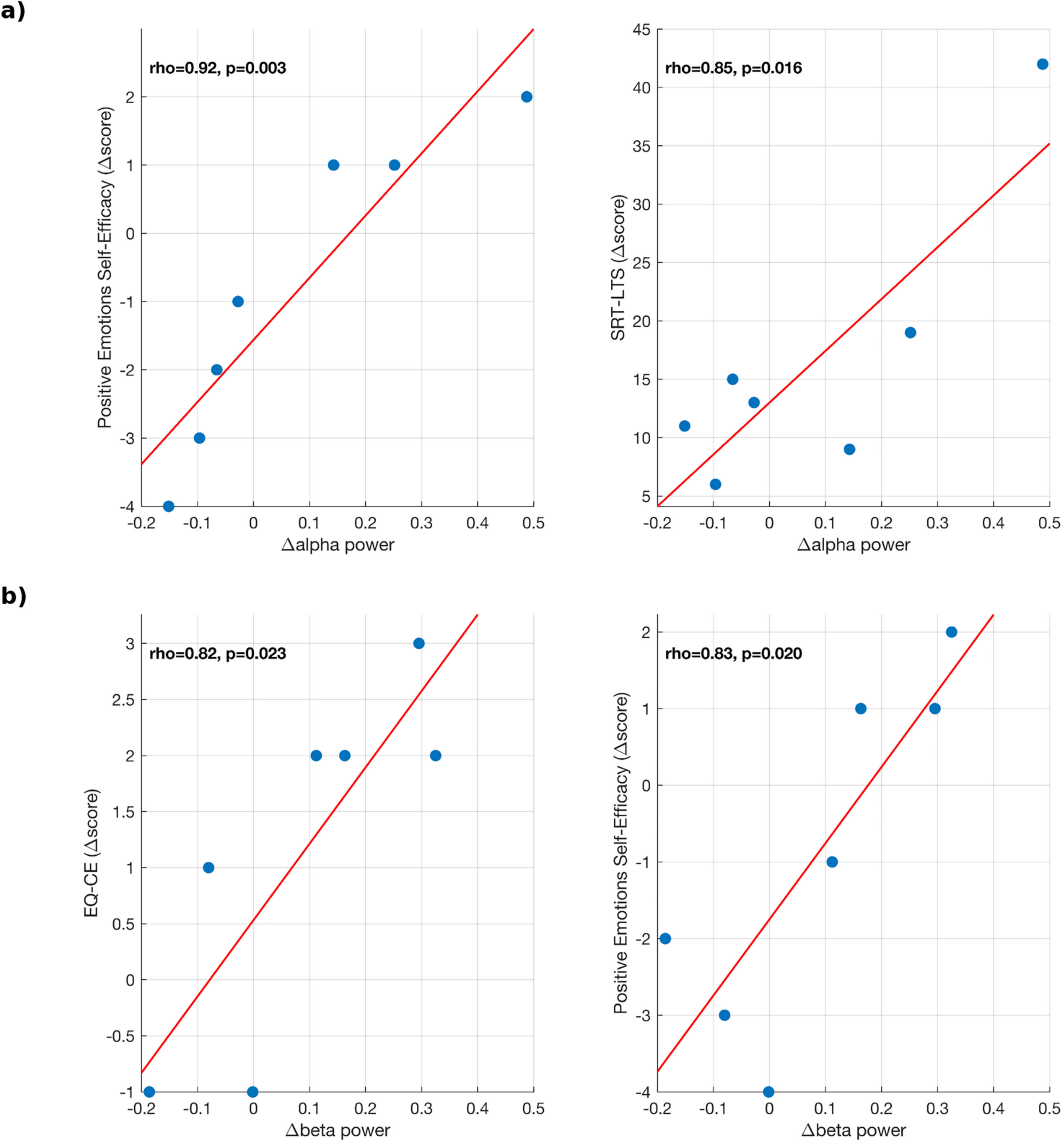
Scatter plots of significant Pearson correlations between EEG power changes and behavioral scores in the MS group for: **(a)** alpha band, **(b)** beta band.

**FIGURE 8:**
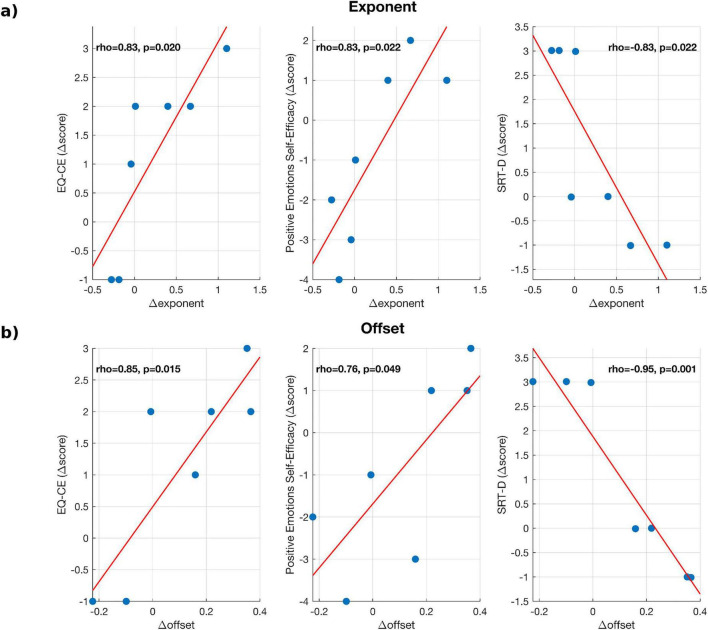
Scatter plots of significant Pearson correlations between differences in aperiodic EEG parameters and behavioral scores in the MS group for: **(a)** exponent, **(b)** offset.

Pearson correlation analyses were performed between both periodic and aperiodic spectral parameters and the neuropsychological scores measuring different features of SC (Tables 6, 7). Increases in alpha and beta power were positively associated with improvements in emotional self-efficacy, balance, memory, and empathy. Specifically, variations in EEG power correlated with the Positive Emotions Self-Efficacy Scale (alpha: *r* = 0.92, *p* = 0.003; beta: *r* = 0.83, *p* = 0.020). In addition, alpha-power changes were associated with better memory performance (SRT-LTS; *r* = 0.85, *p* = 0.016), whereas beta-power changes correlated with increased cognitive empathy (EQ-CE; *r* = 0.82, *p* = 0.023). When considering aperiodic parameters, both the exponent and offset showed significant associations with social-cognitive measures. Higher values of these parameters were positively correlated with cognitive empathy (EQ-CE; exponent: *r* = 0.83, *p* = 0.020; offset: *r* = 0.85, *p* = 0.015) and emotional self-efficacy (exponent: *r* = 0.83, *p* = 0.022; offset: *r* = 0.76, *p* = 0.049), whereas negative correlations were found with verbal memory performance (SRT-D; exponent: *r* = −0.83, *p* = 0.022; offset: *r* = −0.95, *p* = 0.001). The complete set of significant correlations is reported in Tables 4, 5 whereas scatter plots with regression lines are illustrated in Figures 7, 8. Notably, EEG power variations were computed as the difference in log-transformed spectral power [Δ log (power) = log (T1) - log (T0)] for each subject. This approach was adopted to normalize the distribution of spectral values and to express changes in relative rather than absolute power. Thus, positive difference values correspond to proportional increases in oscillatory power, whereas negative values indicate relative decreases. On the other hand, behavioral scores, exponents, and offsets were analyzed on their original linear scale. Overall, these findings suggest that both oscillatory (periodic) and background (aperiodic) neural features are linked to social-cognitive functioning in MS. Specifically, higher alpha/beta power and increased aperiodic offset or exponent values appear to be associated with better emotional, cognitive, and empathic performance. However, given the small sample size, correlation analyses were not corrected for multiple comparisons and should be interpreted as exploratory.

**TABLE 6:** Significant Pearson correlations between EEG power differences (T1–T0) and changes in neuropsychological scores in the MS group.

Band	Test name	*R*	*p*-value
alpha	Positive Emotions Self-Efficacy Scale	0.92	0.003
alpha	SRT-LTS	0.85	0.016
beta	EQ-CE	0.82	0.023
beta	Positive Emotions Self-Efficacy Scale	0.83	0.020

SRT-LTS, Selective Reminding Test —Long-Term Storage; EQ-CE, Empathy Quotient- Empathic Concern.

**TABLE 7:** Significant Pearson correlations between differences (T1-T0) in aperiodic EEG parameters (exponent and offset) and changes in neuropsychological scores in the MS group.

Aperiodic parameter	Test name	*r*-value	*p*-value
Exponent	EQ-CE	0.83	0.020
Exponent	Positive Emotions Self-Efficacy Scale	0.83	0.022
Exponent	SRT-D	−0.83	0.022
Offset	EQ-CE	0.85	0.015
Offset	Positive Emotions Self-Efficacy Scale	0.76	0.049
Offset	SRT-D	−0.95	0.001

EQ-CE, Empathy Quotient- Empathic Concern; Positive Emotions Self-Efficacy Scale; SRT-D, Selective Reminding Test—Delayed.

## 4 Discussion

In the present pilot study, we leveraged clinically available resting-state EEG data to evaluate cortical neurophysiological signatures of MS patients at baseline and after undergoing an 8 week-long VR-rehabilitation program using the CAREN system (Cellini et al., 2022; Maggio et al., 2023, 2024a,2024c). We administered a comprehensive neuropsychological battery targeting multiple facets of SC before and immediately after the rehabilitation period, to examine whether spectral features covaried with these behavioral measures. In addition, we enrolled seven sex- and age-matched healthy individuals as a control group to assess cross-sectional differences in spectral parameters.

To contextualize neurophysiological findings within a clinically meaningful framework, we examined behavioral outcomes associated with the VR intervention. From a clinical perspective, longitudinal analyses revealed significant improvement only in verbal long-term memory (SRT-LTS). Conversely, all other cognitive, emotional, and motor measures remained stable after immersive VR training. The interpretation of these findings should also take into account key clinical characteristics of the MS sample. All participants were diagnosed with relapsing-remitting MS and were in a clinically stable phase, receiving disease-modifying therapies such as ocrelizumab, fingolimod, or natalizumab. Although these treatments are primarily aimed at reducing inflammatory activity and disease progression, they may also indirectly influence cognitive functioning and neural dynamics, potentially modulating responsiveness to rehabilitation interventions. Furthermore, the lack of significant changes across several outcome measures may be related to the relatively short duration and intensity of the intervention, in addition to the pilot nature of the study and the limited sample size. While the protocol (three sessions per week for 8 weeks) is consistent with previous VR-based interventions, it may not be sufficient to induce detectable changes across all cognitive and socio-emotional domains, particularly in clinically stable patients. These findings suggest that longer or more intensive interventions may be necessary to produce more robust and widespread effects. Given the limited sample size (*n* = 7), the absence of statistical significance was anticipated; moreover, reliance on p-values alone may underestimate treatment-related effects in exploratory cohorts. Therefore, the interpretation of T0-T1 changes was supplemented using MCID thresholds, which offer a clinically sound framework for assessing whether observed changes are meaningful from a functional perspective (Draak et al., 2019). Applying MCID thresholds revealed changes indicative of potential functional relevance. The increase in SRT-LTS exceeded the MCID for verbal learning in MS patient cohorts, indicating clinically meaningful improvements in long-term memory consolidation. Working memory also showed clinically consistent improvements, with PASAT-2 and PASAT-3 scores falling within the 10–15% improvement range typically considered significant in MS. Visuospatial memory (SPART) improved by more than 25%, supporting a consistent cognitive trend. In the motor domain, BBS scores increased by almost three points, approaching the established MCID of 3–4 points for balance recovery. Socio-emotional measures showed a similar trend. Cognitive empathy (EQ-CE) improved by approximately 25%, a magnitude considered clinically relevant in MS due to the sensitivity of empathic processes to disease-related alterations. The TAS-20 subscales showed small but directionally consistent improvements in emotion identification and description, while emotional self-efficacy scores remained stable. Overall, these results could indicate that VR-based rehabilitation may have the potential to induce subtle but clinically significant multidomain effects, even in the absence of statistical significance due to sample constraints. Importantly, these behavioral patterns were supported by converging neurophysiological evidence. Increases in alpha and beta power correlated with improvements in emotional self-efficacy, balance, memory, and cognitive empathy, suggesting that oscillatory dynamics may capture early functional changes not fully detectable by group-level statistics. However, these associations should not be interpreted as evidence of a causal relationship or direct clinical improvement, but rather as preliminary correlations that may reflect potential neurophysiological markers associated with socio-cognitive functioning in MS. The alignment between MCID-level improvements and EEG spectral changes could support the hypothesis that immersive VR may be associated with concurrent changes. However, these findings should be interpreted as preliminary associations rather than direct indicators of clinical improvement, and their clinical relevance will require confirmation in larger and adequately powered studies. This convergence supports the rationale for future large-scale, adequately powered clinical trials. Collectively, these multidomain trends suggest that immersive VR may exert subtle yet clinically meaningful effects. This finding aligns with recent evidence showing that VR-based rehabilitation can enhance motor, cognitive, and emotional functioning in neurological populations (Maggio et al., 2019, 2024a; Marotta et al., 2022; Impellizzeri et al., 2024), and extends this body of research by suggesting potential benefits also in socio-affective domains, an area that remains markedly underexplored in MS. One possible explanation is that immersive VR environments could promote experience-dependent neuroplasticity by repeatedly engaging distributed neural networks involved in attention, emotional processing, and social cognition. Through multisensory stimulation, embodied interaction, and task-oriented feedback, VR training could facilitate functional reorganization within fronto-limbic and temporoparietal circuits frequently affected in MS.

Beyond longitudinal changes, cross-sectional comparisons revealed marked differences in baseline oscillatory dynamics between MS patients and healthy controls. Healthy controls were included only as a baseline reference group for EEG spectral characteristics and did not undergo the VR intervention or longitudinal assessments. Therefore, these comparisons should be interpreted as reflecting disease-related neurophysiological features rather than the effects of the VR intervention. Specifically, we found that periodic spectral parameters differed between MS patients and healthy individuals. In particular, our results showed decreased alpha power, especially over fronto-centro-parietal channels in MS patients compared with healthy controls. No significant differences were observed in beta power or in the aperiodic parameters (exponent and offset). These differences were already present at baseline and did not significantly change after the rehabilitation program, suggesting that they likely reflect disease-related neurophysiological characteristics rather than VR-induced effects. Moreover, we observed significant positive correlations between alpha and beta power and measures of social cognition, suggesting that spectral parameters may represent potential neurophysiological markers associated with socio-cognitive functioning in MS, a domain that remains relatively underexplored.

Our finding of decreased alpha power within relapsing-remitting MS patients is in line with previous studies. Decreased alpha power, together with increased theta-power over fronto-central electrodes, has been described in early EEG studies performing power-analysis to detect neurophysiological abnormalities among MS patients (Facchetti et al., 1994; Babiloni et al., 2016a). In addition, coherence within the alpha band was found to be widespread, decreased across MS patients, specifically among cognitively impaired patients with progressive MS (Leocani et al., 2000a). Decreased alpha power has been further observed on timeseries obtained by clinically available (19-channels) EEG systems both at scalp level and at source-level (Babiloni et al., 2016b). Finally, a recent network-level EEG study described lower alpha power in relapsing-remitting SM patients; such spectral property was coupled with abnormalities of network topology (Shirani and Mohebbi, 2022). Taken together, these studies corroborate previous findings in both relapsing-remitting and secondary-progressive MS suggesting that alteration may occur independently of disease phenotype and represent a stable marker of cortical dysfunction in the MS population. A trend of lower alpha power among healthy subjects when compared to MS subjects was also observed in a recent magnetoencephalography study (MEG) though not reaching statistical significance (Van Schependom et al., 2021).

To the best of our knowledge, this is the first study on resting-state EEG data employing spectral parameterization to investigate differences in periodic and aperiodic spectral parameters among MS patients, further supporting previous findings on the alpha band found on MS patients. Spectral parameterization is a relatively novel approach enabling the characterization of aperiodic features of EEG signal together with more investigated oscillatory phenomena (Voytek et al., 2015). During the last decades the aperiodic component (or 1-over-f like activity) has gained more attention in the scientific community, as a consequence, the field is rapidly shifting from one paradigm conceiving aperiodic activity as “neural noise” to a framework where it is considered proper brain-derived signal holding physiological meaning (Gao et al., 2017). Indeed, different computational approaches allow for characterization of both periodic and aperiodic features of power spectra (Voytek et al., 2015), and their spreading across the community has gained insights on how aperiodic parameters change with demographic variables such as age (Voytek et al., 2015), brain states (i.e., wakefulness vs. sleep) (Kozhemiako et al., 2022), cognitive tasks (Waschke et al., 2021), as well as in a plethora of neuropsychiatric conditions for diagnostic or prognostic purposes (Donoghue et al., 2009; Voytek et al., 2015). While parameters describing 1-over-like activity might provide information on excitation/inhibition ratio or shifts in broadband power, it is important to underline that controlling for changes of the aperiodic component is fundamental to properly interpret findings concerning narrow-band oscillations. Specifically, traditional approaches quantify the absolute peak power within pre-defined frequency bands, thus implicitly conflating the contribution of periodic and aperiodic components of the power spectrum (Donoghue et al., 2009). However, changes in the narrow-band peak power or peak frequency might be underlied by: (i) real changes in oscillatory power/peak frequency or (ii) changes of aperiodic features (e.g., increased/decreased exponent or offset), (iii) both the aforementioned scenarios. Consequently, traditional power analyses or calculation of spectral ratio might be potentially flawed by such intrinsic limitations. In this investigation, we did not observe changes in either aperiodic exponent or offset between groups; alpha power, on the other hand, was measured over and above the aperiodic component. Hence the reduction in alpha power observed here not only confirms the results of previous studies but also corroborates the hypothesis that what has been described previously were changes of “pure” oscillatory power rather than changes in the aperiodic component.

Even though this is the first resting-state EEG study to employ spectral parameterization, few recent studies employed the same technique to parameterize power spectra obtained from MEG data. Specifically, Akbarian and colleagues quantified the spectral exponent and beta power over and above the aperiodic exponent in two MS patients subsets: one treated with benzodiazepines and the other without benzodiazepines, finding steeper slopes and higher beta power in the former group as one would expect from clinical pharmacology (more inhibition over excitation and rapid activity in the beta-band over frontal sensors) (Akbarian et al., 2023). Interestingly, in the same study, cognitively impaired MS patients showed a flatter spectral slope in patients when compared to cognitively unimpaired MS patients and healthy control. The results of this study are important for two main reasons; the first one is that the authors mainly focused on quantifying the spectral exponent, bandpass filtering data between 20 and 45 Hz, thus leaving investigation of alpha oscillation out from the analysis. In this scenario, our study constitutes the first demonstration of increased alpha power over and above the aperiodic component; however, this result is to be further replicated in further studies using bigger sample sizes. The second reason is that the increased exponent was observed only among the cognitively impaired MS patients. This could potentially explain why we failed to observe changes in the aperiodic exponent in our cohort, which consists of cognitively unimpaired patients. For this reason, the way both alpha power over and above the aperiodic component and spectral slope relate to cognitive status or in patients SM should be further tackled by future investigations, together with other spectral parameters such as alpha peak-frequency (Finley et al., 2024).

The neurobiological underpinnings of the observed lower alpha power in MS are not entirely clear at the present time. According to Babiloni et al. such narrow-band decrease in the alpha power, described as in our study, during eyes-closed condition (i.e., a state characterized by relative relaxation when subject are less in-touch with the external environment) might be speculatively explained by a desynchronization of diffusion neural networks regulating arousal (Babiloni et al., 2016b). Such hypothesis is further corroborated by results from a large, multi-centric study employing rs-fMRI to analyze network-level dysfunction in various phenotypes of patients with MS (Carotenuto et al., 2022). Specifically, the authors described reduced degree centrality within the saliency and sensorimotor networks with concomitant increase of centrality in default mode network; such alteration were already evident in patients affected by the clinically isolated syndrome (CIS) and relapsing-remitting MS, while being worse in secondarily and primarily progressive forms of the disease (Carotenuto et al., 2022). Interestingly, such network alterations were more severe when comparing cognitively impaired patients versus cognitively unimpaired patients. It might be hypothesized that cortical structural/functional alterations of MS patients lead to hypoconnectivity within ventral attention/salience network while favoring activity in networks that are more active during task-free conditions such as default-mode network (Yeo and Heisler, 2012).

We found positive correlations between alpha and beta power and scores measuring different features of the social cognition; specifically, changes in alpha power were positively correlated with Emotions Self-Efficacy Scale and RAO SRT–LTS. How different aspects of social cognition relate to neurophysiological parameters have been under-investigated in the literature. The complex behavioral patterns that subserve social cognition in a naturalistic environment and their corresponding neural underpinnings have been investigated despite the inherent challenge of the oversimplistic nature of the tasks employed in the neuroimaging field. Interestingly, in a study investigating brain areas involved social exclusion using fMRI, the brain networks involved in mentalization and social pain were respectively default-mode-network and salience network (Schmälzle et al., 2017). It could be hypothesized that neural networks accountable for sustained attention and mind-wandering that have been found to be dysfunctional across MS patients (Carotenuto et al., 2022) might be spatially overlapping with those subserving SC domains such as empathy and theory of mind. However, these speculations should be taken with a grain of salt since the paucity of the studies available on clinical populations (including MS), joining specific neuropsychological texting with neurophysiological/neuroimaging prevents us translate concept that might be valid on healthy individuals to this patient’s group. The correlations here described might be indicators of good outcome and the starting point for future investigations aimed at specifically investigating how VR-rehabilitation, with its multi-sensorial inputs, might shape spectral parameters that are potentially predictive of social cognition among MS patients.

## 5 Limitations

The present study comes with some limitations. First, the small sample limits the statistical power of the analyses and reduces the generalizability of the findings. Although multiple comparisons were controlled using FDR correction, the limited number of participants may still affect the stability of the statistical estimates. Therefore, the reported associations reflect the exploratory nature of this pilot investigation, and the results should be interpreted with caution and considered preliminary. While recognizing it as a limitation we believe that this pilot study might foster new investigations on bigger sample sizes as well as using more advanced EEG recordings (64-channels EEG data, or high density EEG from 128 to 256 channels) which would, on one hand, provide statistical power necessary to make stronger inferences while allowing, on the other hand, for more advanced analyses, as higher spatial resolution would raise the possibility of perform source based analysis or spatial filter to investigate functional connectivity.

In this study, we performed spectral parameterization (Donoghue et al., 2009) a novel technique which interprets EEG signals as a linear combination of periodic and aperiodic features that can be separately measured. However, this approach requires a priori selection of a set of parameters that allow detection of spectral peaks as well as individuation of features of the aperiodic component (i.e., fitting the aperiodic component with or without a knee). Currently, there is no consensus on the set of parameters to be employed when performing spectral parameterization. Here, we employed a set of parameters that have been already employed in the available literature on clinical populations (see Donoghue et al., 2009 for an extensive review), constraining the maximum number of peaks to two, as we were mainly interested to investigate power within alpha and beta bands.

## 6 Conclusion

In conclusion, rehabilitation via immersive VR could represent a promising approach for addressing cognitive, emotional, and motor domains in MS, with preliminary support from EEG analysis findings. This pilot study suggests the feasibility of combining immersive VR with EEG spectral parameterization to study neurophysiological markers associated with sociocognitive functioning in MS. However, given the small sample size, these results should be interpreted with caution. Larger, adequately powered studies and predictive modeling approaches will be required to confirm these findings and clarify the clinical utility of oscillatory and aperiodic EEG parameters as potential biomarkers of treatment response in VR. Future studies should also include larger and more heterogeneous cohorts and adopt longitudinal or randomized controlled designs. In addition, the integration of multimodal neuroimaging approaches, such as high-density EEG or MRI-based techniques, could help further clarify the neural mechanisms underlying VR-related changes in SC.

## Data Availability

The raw data supporting the conclusions of this article will be made available by the authors, without undue reservation.
